# Risk Factors of SARS-CoV-2 Infection: Global Epidemiological Study

**DOI:** 10.2196/28843

**Published:** 2021-08-26

**Authors:** William Alphonse Barletta

**Affiliations:** 1 Department of Physics Massachusetts Institute of Technology Cambridge, MA United States

**Keywords:** COVID-19, pandemic, public health, mortality, infection, risk, risk factors, age, epidemiology, infectious disease

## Abstract

**Background:**

Since the first recognition of the pandemic characteristics of SARS-CoV-2 infection, and before substantial case fatality data were available worldwide, public health agencies warned the public about the increased dangers of SARS-CoV-2 to persons with a variety of underlying physical conditions, many of which are more commonly found in persons over 50 years of age or in certain ethnic groups.

**Objective:**

To investigate the statistical rather than the physiological basis in support of the abovementioned warnings, this study examines correlations globally on a nation-by-nation basis between the statistical data concerning COVID-19 fatalities and the statistics of potential comorbidities that may influence the severity of infection.

**Methods:**

This study considers the statistics describing the populations of the 99 countries with the greatest numbers of SARS-CoV-2 infections at the time of the data cutoff. As national compilations of direct measures of immune system strength are not publicly available, the frequency of fatalities in those countries due to a variety of serious diseases is used as a proxy for the susceptibility of those populations to those same diseases.

**Results:**

The analysis produces plots and calculations of correlations and cross-correlations of COVID-19 case fatality rates and the risks of other potential cofactors. It exposes some reasons that may underlie the degree to which advanced age increases the risk of mortality of infection with SARS-CoV-2. In contrast with the strong influences of comorbidities on the seriousness of consequences of influenzas and their associated pneumonias, the correlations of the same set of risk factors with SARS-CoV-2 infection are considerably weaker. The general characteristics of the observed correlations strengthened through 3 cycles of analysis, starting in September 2020. The strongest correlations were with chronic kidney disease and coronary disease (approximately 0.28 and 0.20, respectively).

**Conclusions:**

This study confirms early clinical observations that infection with SARS-CoV-2 presents an increased risk to persons over the age of 65 years. It does not support the suggestions presented by government agencies early in the pandemic that the risks are much greater for persons with certain common potential comorbidities.

## Introduction

### Background

Only a few months after its disclosure by Chinese health authorities, SARS-CoV-2 had spread worldwide. By late winter of 2020, the World Health Organization (WHO) had designated the disease caused by the virus, COVID-19, to be a worldwide epidemic [1]. As can be seen from the effects of the approximately 80 million infections reported by the end of 2020, COVID-19 can manifest as mild, influenza-like symptoms or, far more seriously, as a severe and often deadly respiratory disease with pneumonia.

From the outset of the COVID-19 pandemic, the public has been exposed to numerous speculations about the degrees to which age and various underlying morbidities may amplify the risk of intensifying the severity of infection with SARS-CoV-2. Authoritative sources such as the US Centers for Disease Control and Prevention (CDC) [2] have issued warnings. Conditions cited by the CDC as increasing risk include cancer, chronic kidney disease, obesity, coronary disease, type 2 diabetes mellitus (DM), and sickle cell disease. The CDC also warns that asthma, hypertension, and liver disease, among other conditions, *may* subject a person to increased risk. In some countries, such as the United States, the incidence of COVID-19 has been more prevalent in some ethnic groups than others [3]**,** leading to speculations that this disparity may be due to biology rather than behavior. Such differences are not unknown; for example, sickle cell disease is most commonly found among persons whose ancestors come from Africa and Mediterranean countries, where malaria is a prevalent affliction [4].

As many of the diseases cited by the CDC are more common in persons in late middle age and older, a common warning early in the pandemic was that SARS-CoV-2 presented a particular danger to persons over 50 years of age. In the initial wave of cases in China [5] and in the strong wave of cases in Italy, the probability of death due to COVID-19 was judged to be a strong function of a patient’s age, being only a few percent for those aged less than 50 years and rising to nearly 20% for patients older than 80 years. The large number of fatalities [6] in care homes in New York, the United Kingdom, and elsewhere have fueled speculations about the risks that comorbidities frequently seen in older people will increase the fatality rate of COVID-19. An alternative explanation is the decrease in immune functions with aging [7].

Why is COVID-19 more dangerous to older than to younger persons? Complicating the answer to this question, the actual mortality rate of COVID-19 remains highly uncertain, as the prevalence of asymptomatic and unreported infections has been estimated to be from 2 to 5 times greater than that of infections with clearly defined symptoms. An early exemplary source of testing-based data was provided by the passengers aboard the Princess Line cruise ship, the *Diamond Princess*, on which half of the passengers who tested positive for COVID-19 were asymptomatic or at least presymptomatic [8]. To some degree, that uncertainty may explain the very wide distributions of reported (or apparent) rates of mortality (case fatality rates) of COVID-19 in different countries, ranging from <0.03% (Singapore) to almost 30% (Yemen). Moreover, in most (but not all) countries, by December 2020, the integrated average case fatality rate had declined significantly from the high levels seen in March and April of 2020.

### Objective

For a less anecdotal (and less speculative) assessment of risk factors for serious consequences of COVID-19, a data-driven examination of worldwide national statistics seems to be in order, with the goal of identifying strong correlations of mortality due to COVID-19 with other potential comorbidities and even with ethnically specific biological and economic factors. Based on a global investigation of the statistical correlations on a nation-by-nation basis between the statistical data concerning reported COVID-19 fatalities and potential comorbidities, this paper presents a set of calculations of linear and multivariate correlations that may influence the severity of the infection.

## Methods

The analysis that follows has not been based on clinical or physiological considerations but rather on national epidemiological statistics as reported to international authorities. Unless otherwise indicated, the following assumptions underlie the subsequent calculations:

1. The *apparent mortality outcomes* (case fatality rates) defined in Equation 1 serve as a *reliable proxy* for actual rates of infection, death, and correlation with comorbidities:



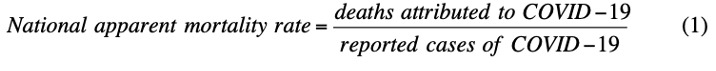



The apparent mortality and case number data used in the following analysis are accurate as of December 30, 2020. This analysis does not and cannot account for any uncertainty due to differing national practices in distinguishing between deaths with COVID-19 and deaths due to COVID-19.

2. The sample of 99 countries across all continents *is representative* of potential correlations between COVID-19 case fatality rates and potential comorbidities or ethnicity. The number of COVID-19 cases in the countries that are not included was not statistically significant at the data cutoff date. Nevertheless, outliers with relatively small statistical significance can skew calculated correlations.

3. *Linear correlations* are examined on the basis of national data for COVID-19 for the year 2020 and comorbidities for the year 2018. The sources that describe the prevalence of disease are the WHO, as reported by World Life Expectancy [9], and Worldometer [10], and the economic data are sourced from the World Bank as reported by Trading Economics [11]. This analysis *assumes* that the published WHO data concerning the fatalities ascribed to diseases in a given country constitute valid proxies for the prevalence of those maladies in national populations. In the case of obesity, the reported number is the percentage of the population with a BMI exceeding a WHO-established standard for a person of that sex.

The study examined the following factors:

- *Demographics:* geographical region, population, and national median age
- *SARS-CoV-2:* number of COVID-19 tests, confirmed cases of COVID-19 as reported by government authorities, and the apparent case fatality rate
- *Medical factors:* incidence of influenza, lung disease, asthma, obesity, heart disease, common cancers, hypertension, chronic kidney disease, diabetes, and malnutrition
- *Economics:* gross domestic product–purchasing power parity (GDP-PPP), average household size, percentage of the population living in slums, health expenditures per capita, and WHO Universal Health Coverage (UHC) index
- *One random (or pseudorandom) variable* in the range from 0 to 100

Examination of the data began with computing linear correlations between variables. The evaluation of the linear correlation herein uses the Pearson product moment correlation (Equation 2) to evaluate linear relationships between data sets:



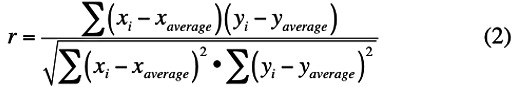



One may estimate the *statistical significance* of calculated correlations by computing *r* for two variables that are *uncorrelated by construction* (ie, apparent COVID-19 mortality and a random variable in the range from 1 to 100). Once linear correlations have been computed, the next step is evaluating cross-correlations among variables and performing a multivariate analysis.

The 99 countries sampled in this study were selected as those reporting the largest numbers of COVID-19 infections. The countries listed in Table 1 represent 5 geographical regions: the Americas, Asia, Europe, Africa, and Middle East plus Central Asia. Regional populations were included. The combined population of nearly 5.5 billion persons accounts for the strong preponderance of all cases reported worldwide. The data cutoff date was December 30, 2020.

The SARS-CoV-2–related data are aggregated by sex because many countries still do not report sex-disaggregated data (or make these data available publicly). Therefore, the frequently reported sex-based disparities in contagion and in the case fatality rate could not be examined with respect to sex-based differences in occurrences of potential comorbidities.

**Figure 1:**
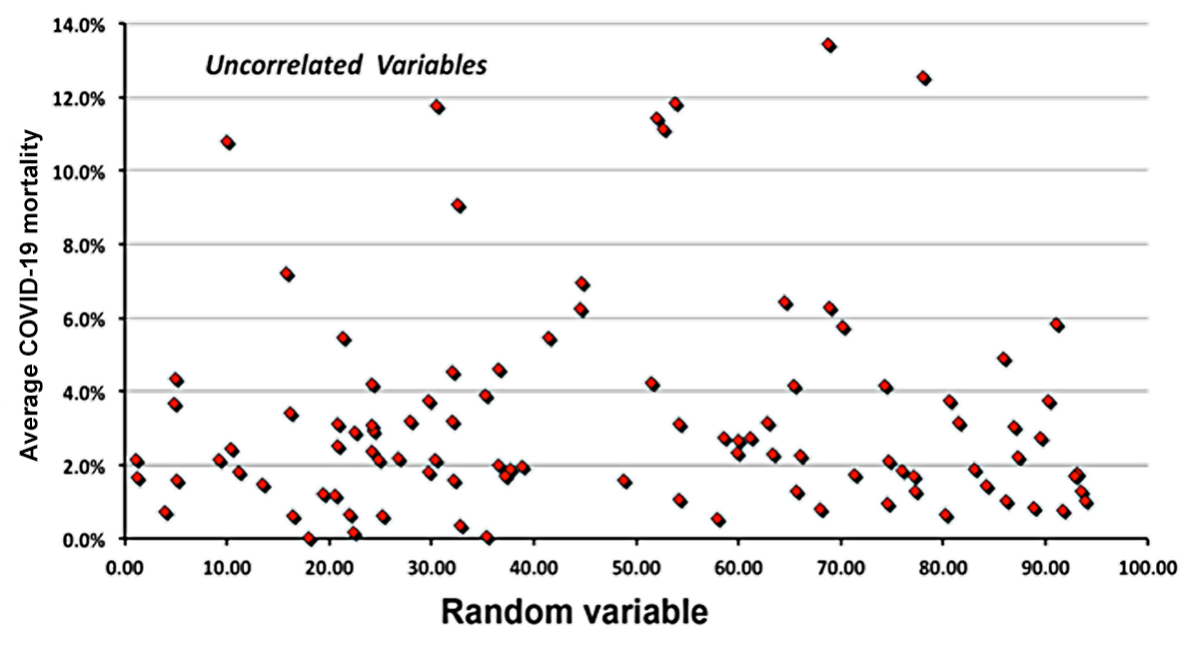
A plot of the variables uncorrelated by construction.

Figure 1 plots the case fatality rates and random numbers that are uncorrelated by construction. The Pearson coefficient for this set of 99 values is 0.053.

A potential limitation of this approach is that all mortality data have equal weight in the calculation of the correlation. One check of whether this ansatz introduces a bias is the correlation between apparent national mortality rates and national populations. Calculating this correlation yields a value of –0.014 which is close to the Pearson coefficient for uncorrelated variables. Another possible way to attribute a rational weighting is to plot the variation of COVID-19 deaths per capita against the possible risk factor. However, the number of fatalities per capita depends strongly on national public health policies, national efforts to prevent the spread of SARS-CoV-2, the GDP, and other nonmedical considerations. The differences in COVID-19 statistics between Norway and Sweden [10] are cases in point.

**Table 1:** Countries sampled grouped into 5 regions. Note that as Yemen is a statistical outlier in apparent mortality, many plots omit its data point for visual clarity.

Region	Population (million)	Countries
Americas	977	Argentina, Bolivia, Brazil, Canada, Chile, Columbia, Costa Rica, Dominican Republic, Ecuador, El Salvador, Guatemala, Honduras, Mexico, Panama, Paraguay, Peru, United States, Venezuela
Asia	2504	Australia, Bangladesh, China, India, Indonesia, Japan, Kazakhstan, Kyrgyzstan, Korea, Malaysia, Nepal, New Zealand, Pakistan, Philippines, Singapore, Thailand, Taiwan
Europe	725	Albania, Armenia, Austria, Azerbaijan, Belarus, Belgium, Bosnia, Bulgaria, Croatia, Czechia, Denmark, Estonia, Finland, France, Germany, Greece, Hungary, Ireland, Italy, Macedonia, Moldova, Netherlands, Norway, Poland, Portugal, Romania, Russia, Serbia, Spain, Sweden, Switzerland, Ukraine, United Kingdom
Africa	768	Algeria, Cameroon, Congo, Ethiopia, Ghana, Ivory Coast, Kenya, Libya, Madagascar, Mali, Morocco, Nigeria, South Africa, Sudan, Uganda, Zambia
Middle East	487	Afghanistan, Bahrain, Egypt, Iran, Iraq, Israel, Lebanon, Kuwait, Oman, Qatar, Saudi Arabia, Turkey, United Arab Emirates, Uzbekistan, Yemen

## Results

### Examination of Linear Correlations

**Figure 2:**
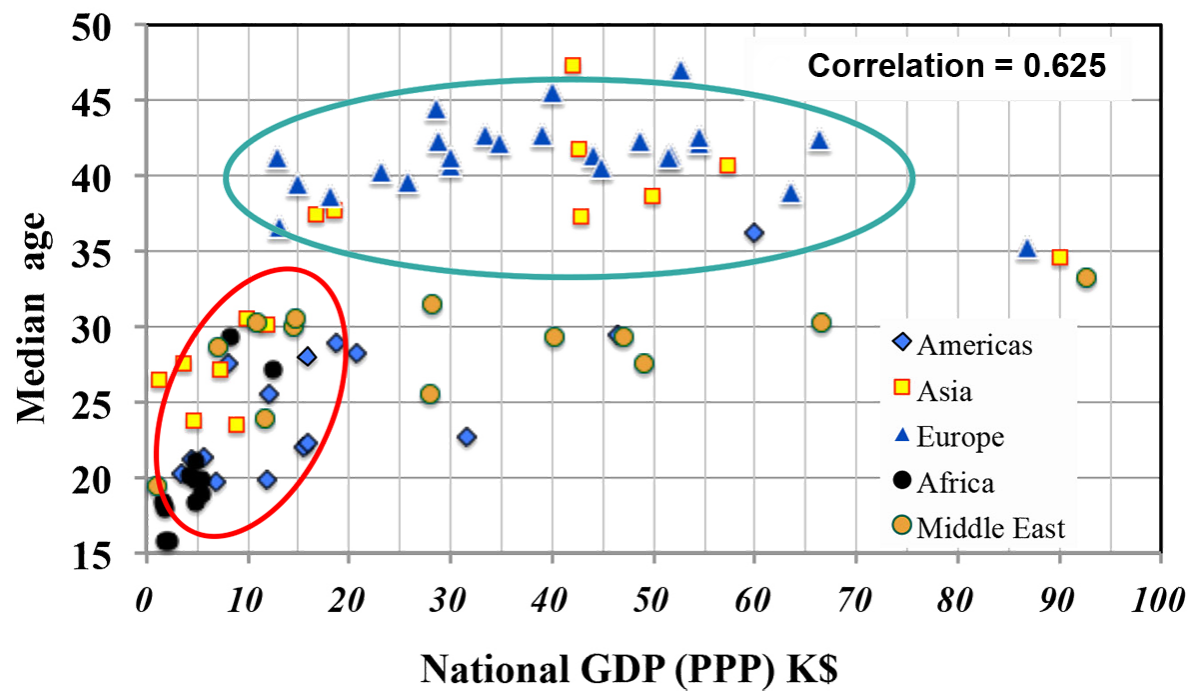
The plot of GDP-PPP corrected for purchasing power versus median age in countries from the 5 regions under study. GDP(PPP): gross domestic product corrected for purchasing power parity; K$: US $1000.

**Figure 3:**
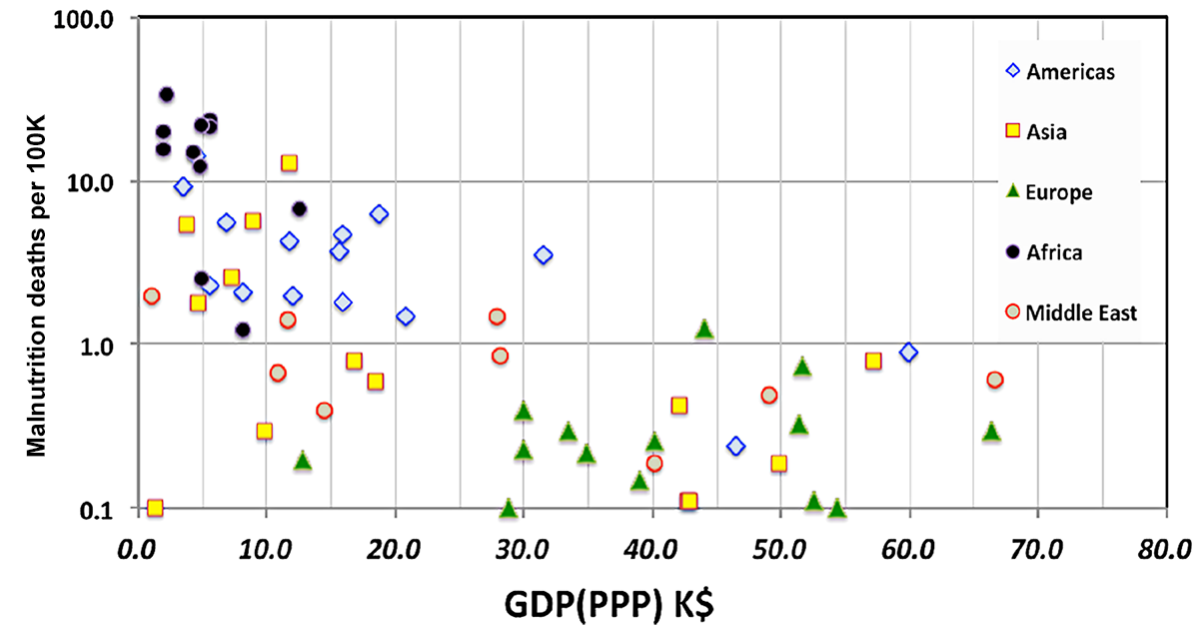
Correlation of poverty with malnutrition. 1K: 100,000 persons; GDP(PPP): gross domestic product corrected for purchasing power parity; K$: US $1000.

To gain confidence in this statistical approach, one can plot two variables for which one may expect to see a correlation: GDP-PPP and median age (Figure 2). Here, the linear correlation is quite high (0.625). Closer examination of Figure 2 suggests a limitation of considering only linear correlations. The countries circled in red show a strong correlation, while those in the green ellipses show scarcely any correlation of a nation’s wealth with the age of its population. Clearly, a refinement of the statistical approach is needed. Identifying the data underlying each point with each country’s region in Figure 3 reveals that median age and national wealth are essentially uncorrelated for European nations but strongly correlated for countries in Africa and Asia. *Regional grouping* was thus adopted throughout this study.

To illustrate the utility of this refinement, Figure 3 shows the correlation between deaths per 100,000 persons due to malnutrition and national wealth measured by GDP corrected for purchasing power (GDP-PPP). The relatively strong (0.455) correlation is driven by the high rates of malnutrition in Africa, Central America, and the less economically advantaged countries of Asia. No such effect is apparent in Europe.

**Figure 4:**
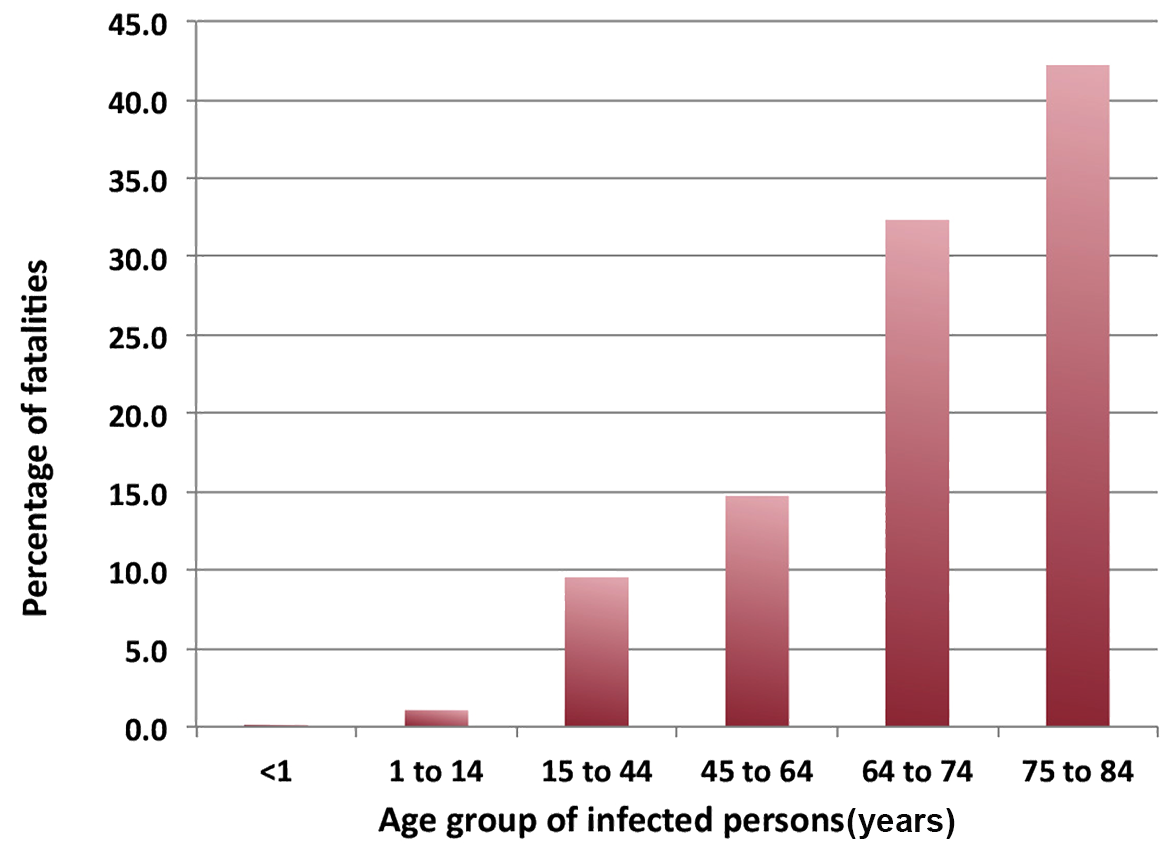
Deaths attributed to COVID-19 by the UK Office of National Statistics [9,10].

From the outset of the pandemic, national health authorities warned the public about the increased risk of mortality for persons 60 years of age and older. Figure 4 shows an early example of the basis for these warnings in the data provided by the UK Office of National Statistics in September 2020 [12] and also in reference [13]. The UK government website notes several caveats: (1) the figures include deaths of nonresidents of the United Kingdom; (2) they are based on the date that a death was registered rather than when it occurred; (3) they are provisional and use the tenth edition of the *International Classification of Diseases* for definitions of the coronavirus (COVID-19). Again, the question arises of why the severity of COVID-19 infection should be a function of age.

**Figure 5:**
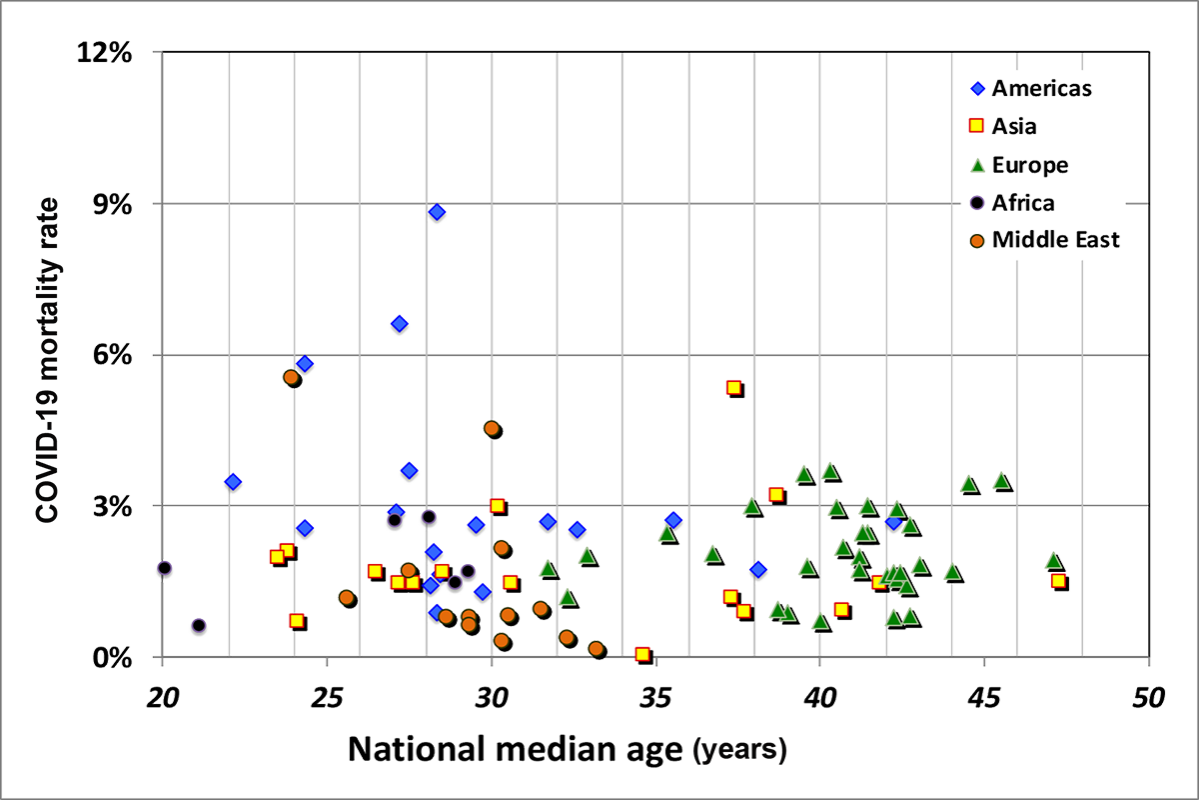
National median age versus case fatality rate for the 5 regions under study.

From these data, one might expect a strong correlation between the apparent national case fatality rate and the median age of a country’s population. Even accepting the hypothesis of universality for the data in Figure 4, one should first multiply these rates by the demographics of a nation’s population normalized to the UK population grouped into the same age bins. Such a plot (Figure 5) shows a surprising result. The overall linear correlation is negative, –0.181, partially due to the disparity among the regions: –0.258 for the Americas, 0.052 for Asia, 0.141 for Europe, 0.02 for Africa, and –0.608 for the Middle East and Central Asia.

Rather than plotting the COVID-19 case fatality rate versus the national median age, one might examine the dependence on the percentage of the population of people aged 65 years or greater. In that case, the overall correlation (–0.081) is negative, consistent with reference [14]; however, this is mostly the result of regional variations, with a larger but still relatively low correlation (approximately 0.19) in Europe and Africa.

**Figure 6:**
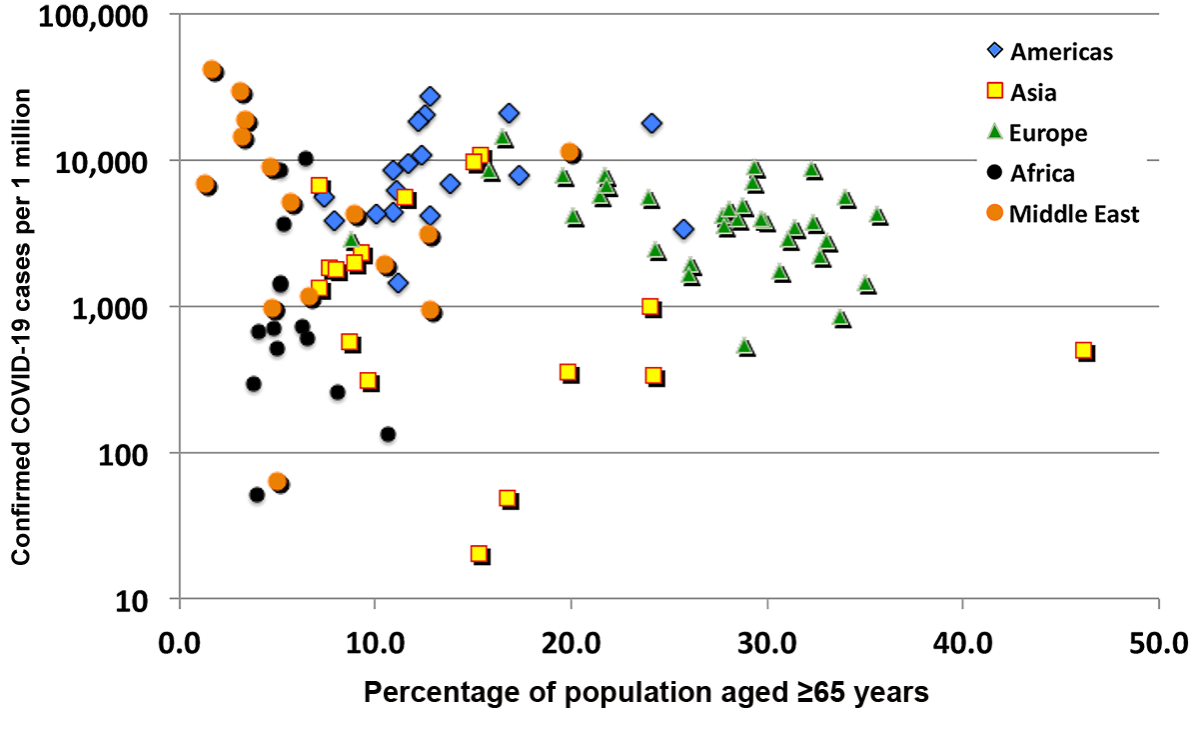
Confirmed COVID-19 cases per 1 million persons as a function of the percentage of the population aged 65 years and older.

As a measure of the influence of the age of a population on SARS-CoV-2 contagion, the national rate of confirmed cases of COVID-19 per 1 million persons with respect to the percentage of the population aged older than 65 years (Figure 6) displays a moderate correlation of 0.447.

**Figure 7:**
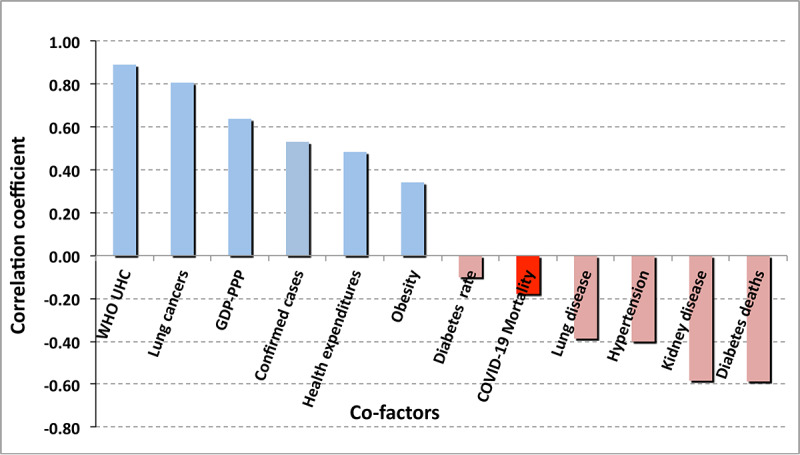
Correlations of potential risk factors with national median age. GDP: gross domestic product; UHC: Universal Health Coverage; WHO: World Health Organization.

One may hypothesize that the “care home effect,” as in, the large numbers of deaths seen in nursing homes in Italy, the United Kingdom, and the US state of New York, was more the result of overcrowding and poor hygienic practices compounded with the general infirmity and the reduced immune function of nursing home residents than of any extreme dependence of the lethality of COVID-19 on individual, specific, underlying medical disorders. The linear correlations of age with potential causal factors, shown in Figure 7, suggest the strength of candidate cofactors to explain the care home effect. In addition to specific cofactors, the care home effect also reflects a generally very weakened physical condition of many occupants of care homes, which could render any pneumonia-inducing disease potentially lethal. The data in Figure 7 show no evidence that age alone influences the probability of a person becoming infected with SARS-CoV-2.

Figure 7 may explain what appears to be a startling result, namely, the globally negative correlation of the COVID-19 case fatality rate with the age of the national population. The negative value is due to the strong correlations between the national median age and the combination of adjusted GDP (0.64), national health care expenditures (0.48), and the WHO Universal Health Care Index. Nations with the oldest populations are generally those that are the wealthiest and in which health care services are the most robust, thus reducing the level of mortality.

**Figure 8:**
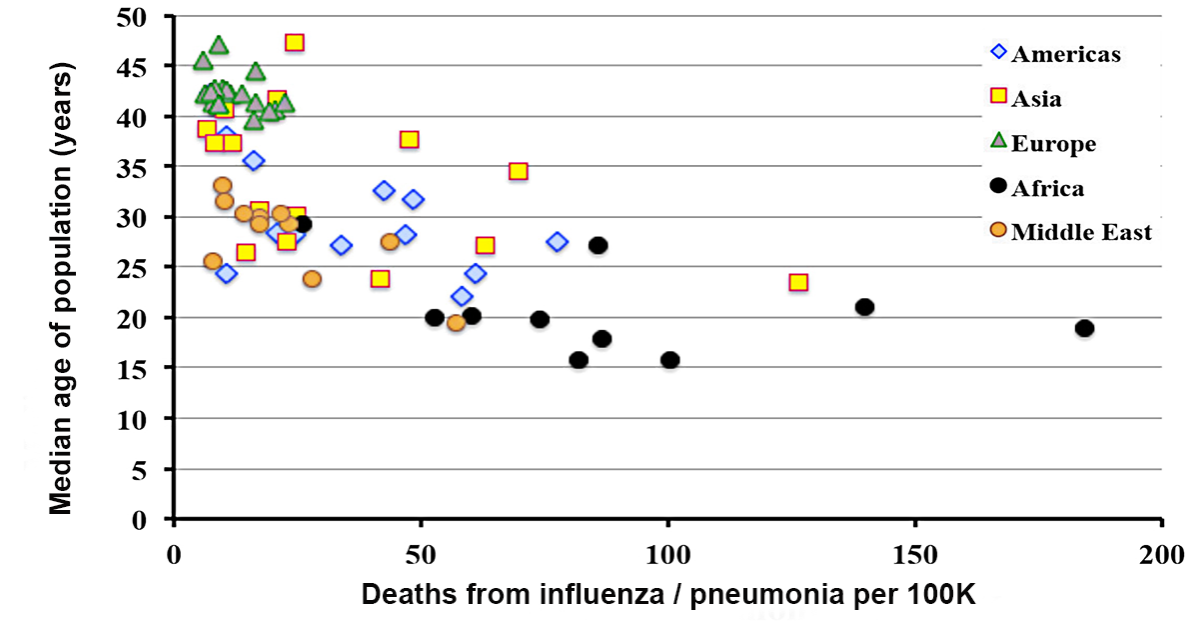
Incidence of influenza-related pneumonia deaths as a function of national median age.

In contrast with infections due to SARS-CoV-2, fatalities from influenza-induced pneumonia (Figure 8) are highly correlated (–0.652) with the median age of the population. The correlation also displays a strong regional dependence. The correlation is negative for the same reasons previously explained for COVID-19.

**Figure 9:**
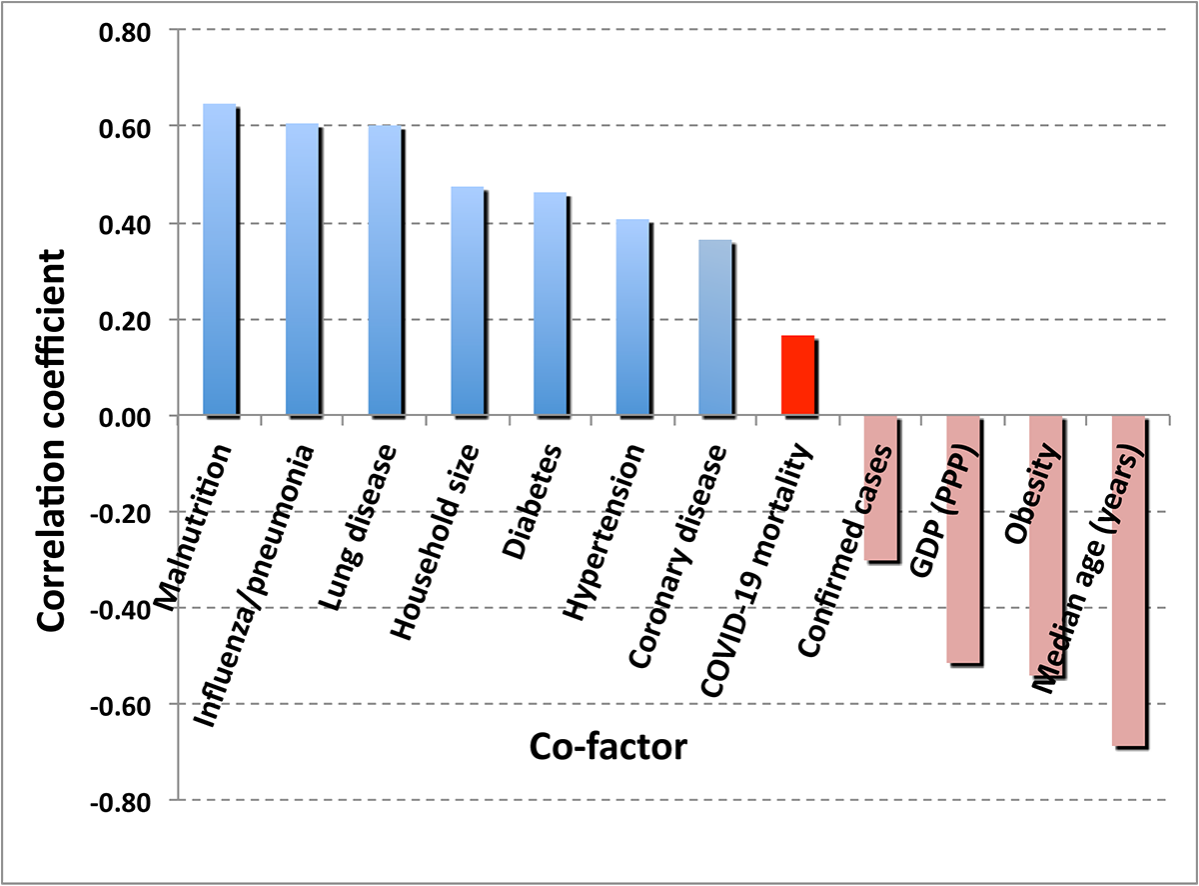
Correlation of severe asthma with the COVID-19 case fatality ratio. GDP(PPP): gross domestic product corrected for purchasing power parity.

This result for influenza suggests the hypothesis that because COVID-19 typically presents as a severe respiratory disease, the severity of COVID-19 infection may correlate with the incidence of asthma. The global value (Figure 9) is small but not negligible (0.165), largely driven by the strong correlation (0.68) in the Middle East.

For asthma as a cofactor, the contrast with influenza-related pneumonia is striking. A relatively high overall correlation of 0.594 for influenza was observed in all regions. Hence, any reference to COVID-19 as a “flu-like” infection or as a “superflu” is grossly misleading.

**Figure 10:**
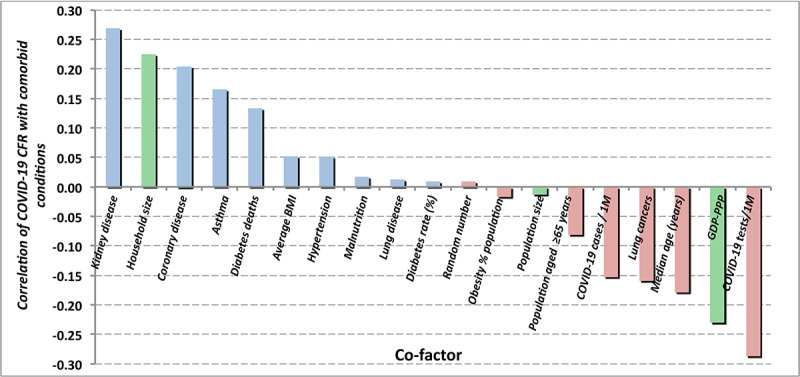
Summary of linear correlations with national COVID-19 case fatality ratio data. 1M: 1 million; CFR: case fatality rate; GDP: gross domestic product.

The CDC issued a warning early in 2020 that obesity represented a comorbidity that could potentially lead to severe consequences of a COVID-19 infection. However, once again, the actual national data (Figure 10) essentially display no correlation (–0.017) of a country’s COVID-19 case fatality rate with the percentage of its population that is considered obese. A better metric of national obesity may be the average BMI (in kg/m^2^) of the population. With BMI as the metric of the national prevalence of obesity, the correlation increases to 0.052, which is still very small. Moreover, that figure may itself be misleading when comparing regions, as the correspondence between BMI and body fat percentage varies considerably from country to country (10% to 20%).

**Figure 11:**
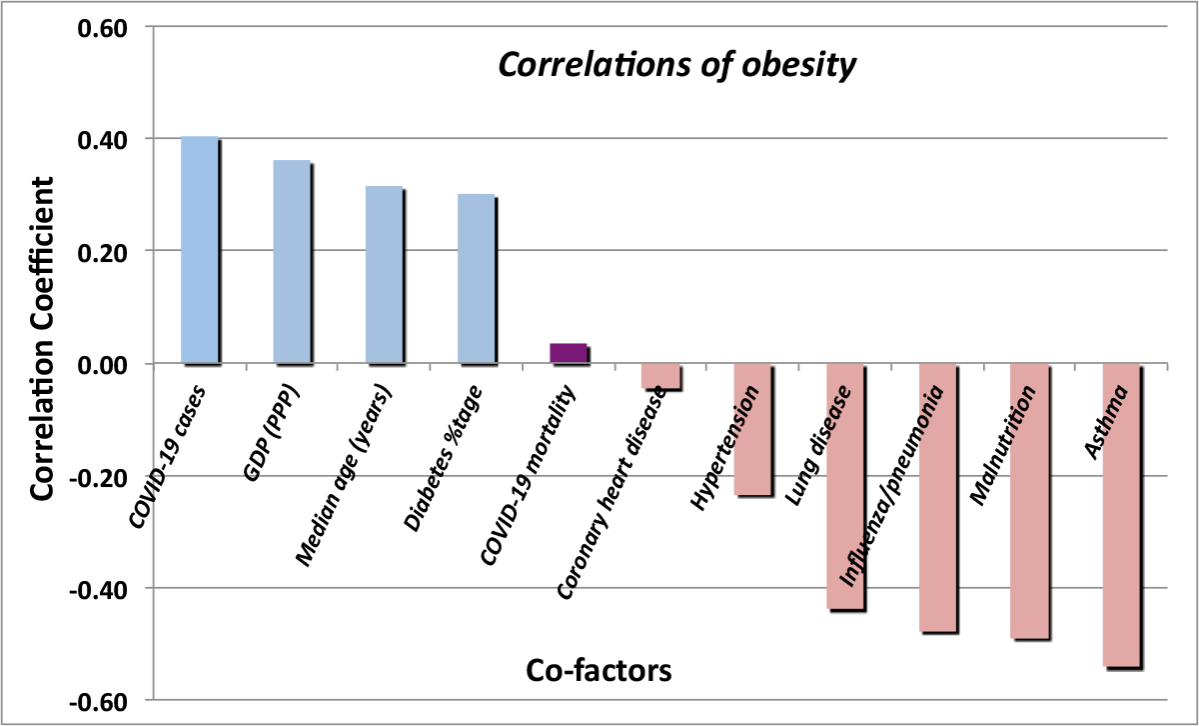
Correlations of obesity rates with COVID-19 mortality and other conditions. For most conditions, rates are based on deaths per 100,000 persons. GDP (PPP): gross domestic product corrected for purchasing power parity.

The contribution of obesity to the *outcome* of other pulmonary disorders is significantly different from that of COVID-19, as is displayed in Figure 11. Obesity does have a significant correlation (0.516) with the risk of contracting SARS-CoV-2 infection, although not with the apparent outcome of the infection. Observation of increased risk of infection (although not its outcome) was previously reported in [13]. Reference [14] reports an increased risk of infection (0.329) for people with chronic kidney disease. That correlation of risk of infection is not seen in the statistics of this study, which consistently found a temporally increasing negative correlation (–0.046). However, as shown in Figure 10, this study does confirm an increased risk of mortality (0.269) for persons with chronic kidney disease who do develop COVID-19.

One might speculate that as a chronic respiratory disorder involving pulmonary airways, asthma would increase the seriousness of the consequences of COVID-19 and its induced pneumonias; this analysis shows no such significant correlation (0.053). Examining the correlation of COVID-19 mortality with other lung diseases also showed a very small correlation (0.013). In contrast, the relationship of influenza-induced pneumonias with asthma and other lung diseases presents correlations that are quite high, 0.594 and 0.348, respectively. With respect to their effects on patients with underlying conditions, influenza and COVID-19 are very different diseases.

Another early warning to persons with underlying conditions concerned DM. That suspicion is echoed by the strong increase of incidence of diabetic conditions with age. Whether one measures the incidence of diabetes by deaths due to diabetes or to the reported national rates of diabetes in adults (20-79 years of age), the correlation with COVID-19 mortality is similarly low (0.109). In *otherwise healthy persons*, diabetes does not appear to be a significant risk factor with respect to the serious complications of infection with SARS-CoV-2.

Figure 10 and Table 2 summarize the linear correlations and their time variations, respectively, of the COVID-19 case fatality rate with underlying medical and economic conditions (green bars in the figure) considered herein. As the percentage of the population over 65 years of age correlates at best weakly with the apparent COVID-19 case fatality rate, one may surmise that poor health care management played a very large role in the care-home effect.

Figure 10 shows a strong negative correlation of the case fatality rate with both COVID-19 tests per million persons and with the number of cases per million persons. More tests mean earlier detection, more detection of mild and weakly symptomatic cases, and better triage followed by earlier and more effective clinical treatments.

**Table 2:** Correlations with national values of apparent COVID-19 case fatality rates.

Potential cofactor	Correlations with COVID-19 case fatality rate statistics by date		
	December 30	November 20	October 16
Kidney disease	0.269	0.289	0.176
Household size	0.225	0.228	0.126
Heart disease	0.204	0.194	0.099
Asthma	0.165	0.168	0.091
Diabetes deaths	0.133	0.148	0.05
COVID-19 deaths per 1 million persons	0.092	0.07	0.17
Percentage of the population living in slums	0.090	0.072	0.059
Hypertension	0.051	0.049	–0.011
Influenza/pneumonia	0.034	0.040	–0.020
Malnutrition	0.017	0.002	–0.037
Lung disease	0.013	0.024	–0.112
Random number	0.009	–0.024	0.026
Percentage of the population with diabetes	0.009	0.046	–0.043
Population	–0.013	0.000	–0.014
Percentage of the population with obesity	–0.017	–0.006	0.014
Percentage of the population aged ≥65 years	–0.081	–0.103	0.028
COVID-19 cases per 1 million persons	–0.153	–0.163	–0.086
Health care expenditure	–0.155	–0.143	–0.02
Lung cancers	–0.159	–0.179	–0.098
Life expectancy	–0.163	–0.152	–0.055
Median age	–0.179	–0.191	–0.074
World Health Organization Universal Health Coverage index	–0.197	–0.168	–0.076
Percentage of the population living in cities	–0.197	–0.177	–0.121
Adjusted gross domestic product	–0.23	–0.215	–0.119
COVID-19 tests per 1 million persons	–0.287	–0.257	–0.111

### Cross-Correlations and Multivariate Analysis

Before investigating cross-correlations for root causes, one should perform a multivariate analysis of the COVID-19 case fatality rate against a common trio of risk factors commonly found in patients in nursing and convalescent homes–namely DM, hypertension, and coronary disease. For that trio, the coefficient of multiple correlation is 0.171, which is not negligible but is unlikely to be the root cause of the care home effect. Computing the correlation of DM, hypertension, and coronary disease with deaths due to influenza and its associated pneumonia yields a stronger correlation of 0.359. Replacing hypertension with asthma in the DM, hypertension, and coronary disease trio reduces the coefficient of multivariate correlation for COVID-19 mortality to 0.121. In contrast, analogous analysis for influenza increases the multiple correlation coefficient to 0.627, demonstrating once again (see Table 3) that influenza and COVID-19 are very different diseases.

Other calculations of multivariate correlations with the apparent national mortality rates of COVID-19 are presented in Table 4.

**Table 3:** Multivariate correlations for a trio of input variables: diabetes mellitus, hypertension, and coronary disease.

Output variable	Regression coefficient	Pearson *r* values		
		Diabetes mellitus	Hypertension	Coronary disease
COVID-19	0.123	0.035	0.053	–0.041
Influenza/ pneumonia	0.439	0.386	0.147	0.247

**Table 4:** Multivariate correlations with national COVID-19 mortality data.

Multiple variables	Regression coefficient	Pearson r values
Gross domestic product and household size	0.07	–0.059, 0.056
Obesity and diabetes	0.035	0.035, –0.071
Influenza and lung disease	0.117	–0.064, –0.148
Diabetes, heart, and hypertension	0.123	0.035, 0.053, –0.041
Median age and number of cases	0.138	0.004, –0.137
Influenza deaths and diabetes	0.107	–0.064, 0.035
Influenza deaths and hypertension	0.068	–0.064, –0.041
Obesity, asthma, and diabetes	0.142	0.035, 0.053, 0.035

### Cross-Correlations

**Figure 12:**
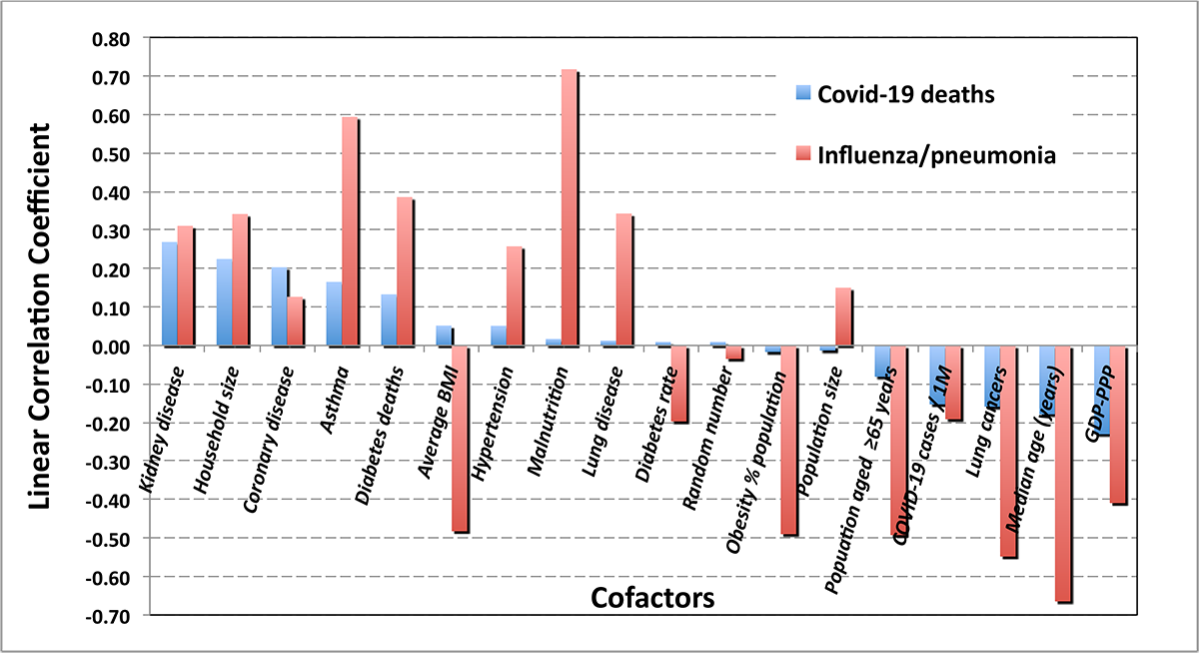
Contrast between correlations of COVID-19 with influenza-induced pneumonia. GDP: gross domestic product.

The previous section argues and Figure 12 illustrates that there is a striking contrast between the correlations of COVID-19 with those of influenza/pneumonia with respect to other potential underlying conditions.

**Figure 13:**
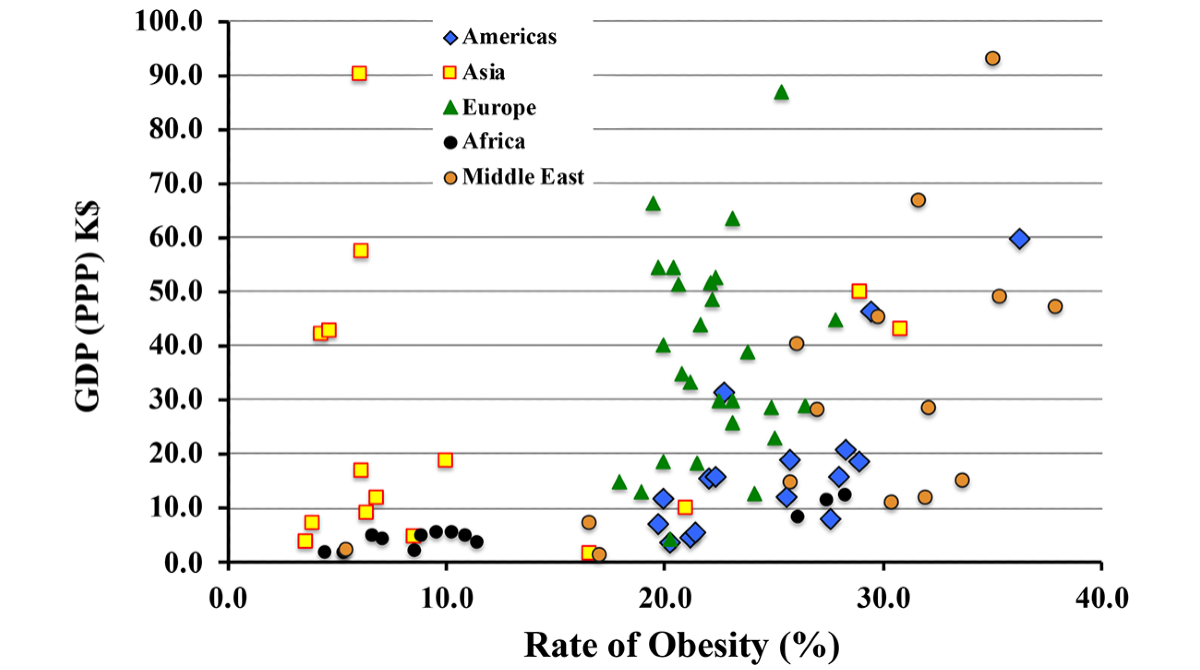
Correlation of regional wealth with obesity. GDP(PPP): gross domestic product corrected for purchasing power parity; K$: US $1000.

Although obesity appears to be correlated with SARS-CoV-2 contagion, it appears uncorrelated with the outcome of COVID-19 infections, contrary to the findings of reference [15]. Understanding the correlations of obesity calls for a deeper look at the relationship of obesity with the conditions that show the most influence. Already, in the case of contagion, regional differences represent a substantial fraction of the apparent effect. The regional differences could be due to factors such as national median age, or they may be influenced by national wealth reckoned in terms of per capita GDP-PPP, as shown in Figure 13.

**Figure 14:**
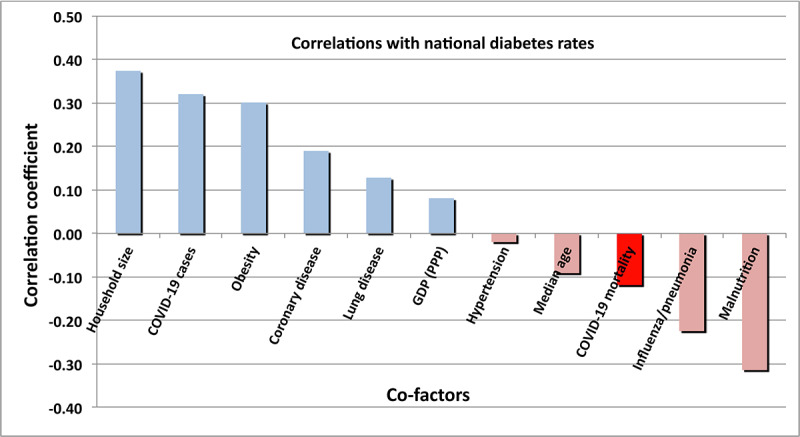
Correlations of national rates of diabetes mellitus with other medical and economic conditions. The red bar represents the correlation with COVID-19 mortality. GDP (PPP): gross domestic product corrected for purchasing power parity.

As is the case with asthma, DM (Figure 14) shows significant correlations with several medical and economic conditions, such as age, household size, and mortality due to influenza/pneumonia. Once again, no correlation with COVID-19 mortality (red bar) is evident.

### Regional Analysis

**Figure 15:**
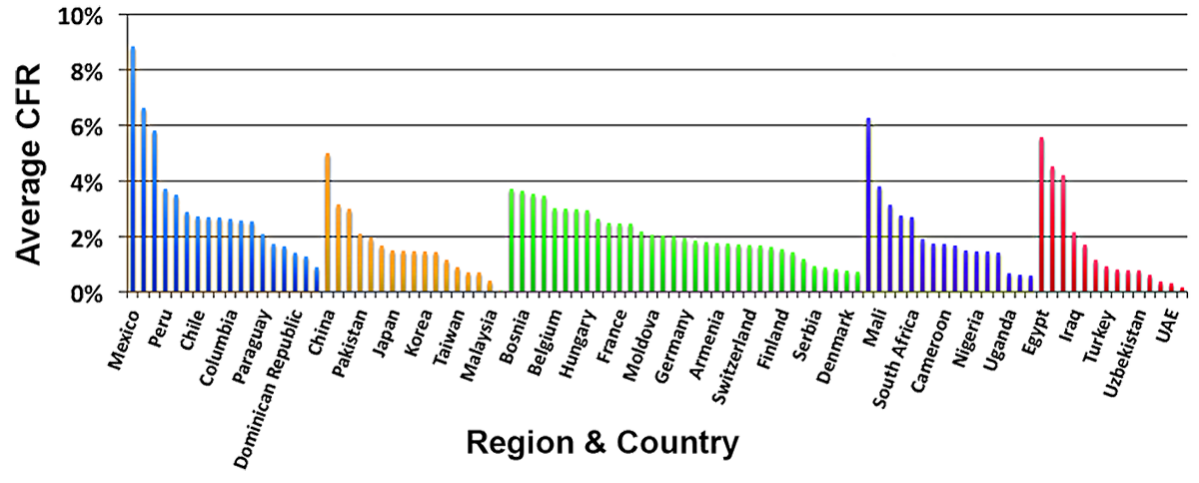
Average case fatality rate by country and region. CFR, case fatality rate; UAE: United Arab Emirates.

A key assumption of this study is the high degree of country dependence of the COVID-19 case fatality rate. Even though the case fatality rate has fallen dramatically in many countries with rates originally greater than 10%, after nearly one year of the pandemic, the disparity by country and by region remains large, ranging over one order of magnitude, as illustrated in Figure 15.

The size of the regional data sets is obviously much smaller and the uncertainties in computed correlations are much higher than those of the aggregated world data. However, examining the regional dependence of the COVID-19 case fatality rates on the most commonly cited comorbidities is instructive. (See Table S1 of Multimedia Appendix 1.)

### Factors Related to National Economics and Public Health Policies

The differences in the magnitudes, outcomes, and characteristics of the waves of infections among national subregions with roughly equivalent medical factors indicates that economics and public health policies makes a significant difference in the severity of SARS-CoV-2 infection. This section examines dependencies on GDP-PPP, average household size, percentage of the population living in slums, percentage of the population living in cities, health expenditures per capita, and the WHO UHC index.

Figure 3 has already shown an example of economic impact on medical outcomes; the per capita GDP-PPP has a strong influence (–0.446) on the rate of deaths due to malnutrition. That observation is hardly surprising. One might also ask whether per capita GDP-PPP would have a similar impact on mortality due to COVID-19 infections. The distribution of COVID-19 mortality with national wealth shows essentially no correlation (–0.059). The politics of poverty does not, of itself, explain the observed national rates of COVID-19 mortality.

**Figure 16:**
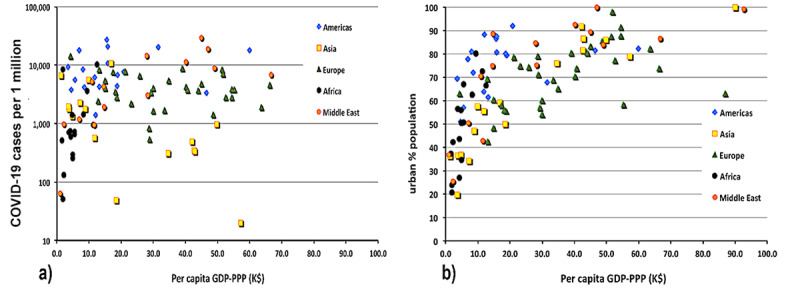
(a) The distribution of COVID-19 cases with national GDP-PPP. (b) The degree of urbanization with increasing GDP. GDP-PPP: gross domestic product–purchasing power parity; K$: US $1000.

The distribution of contagion of SARS-CoV-2 over the global data set is noticeable and positive (0.299). However, as shown in Figure 16, that value is entirely driven by the strong dependence of the rising contagion on rising income in African countries. If one removes the African countries from the sample, the correlation disappears (0.028).

The differing behavior in Africa, which can be observed in Figure 16 (a), may be due to the increase in urbanization with increasing national wealth. One might further suspect that the increase in urbanization is also likely to increase the fraction of the national population living in slums. In fact, the percentage of people living in slums actually decreases with the urbanized fraction of the population.

The correlation of economic and policy factors with contagion (measured in confirmed COVID-19 cases per 1 million persons and apparent COVID-19 mortality) is presented in Table 5. As the mortality rate varies in time and seems to decline as the pandemic progresses (at least in the northern hemisphere), the mortality rate was benchmarked on December 30, 2020. The surprising negative correlation in contagion with the percentage of the urban population living in slums is likely due to the trend in Africa that the smaller the fraction of the population living in cities, the more likely it is that they live in slums (World Bank data).

**Table 5:** Correlations of economic and political factors with numbers of cases of COVID-19 infection (contagion) and apparent COVID-19 mortality.

Factor	Correlation	
	Contagion	Mortality
Percentage of population living in cities	0.476	–0.085
Testing for COVID-19	0.438	–0.099
Gross domestic product–purchasing power parity	0.32	–0.059
World Health Organization Universal Health Coverage index	0.303	–0.02
Health spending	0.24	0.046
Household size	0.172	0.056
Percentage of urban population living in slums	–0.407	0.041

**Figure 17:**
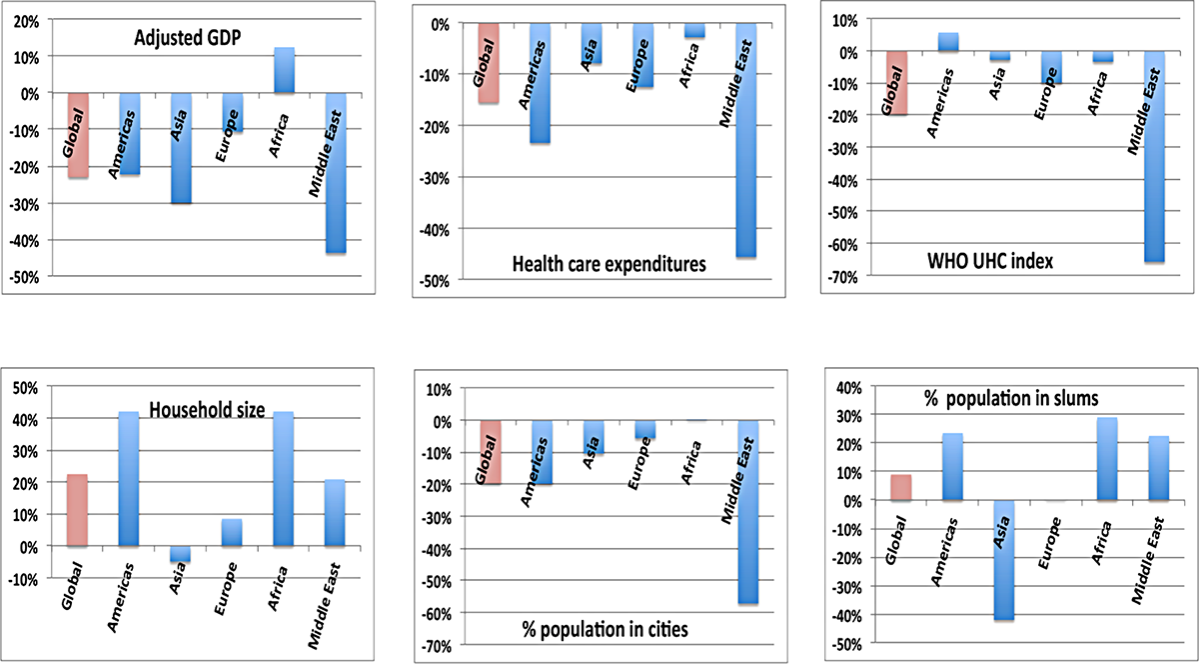
The impact of economic cofactors on case fatality rates, showing a strong variation by region. GDP: gross domestic product; UNC: Universal Health Coverage; WHO: World Health Organization.

An examination by region of the impact of economic cofactors in the COVID-19 case fatality rate is shown in Figure 17. The negative correlations with national wealth and with national health care expenditures are to be expected. Nonetheless, these effects are weaker in Africa than in other regions. More detailed investigation of these effects would require examination of underlying conditions on a country-by-country basis.

**Figure 18:**
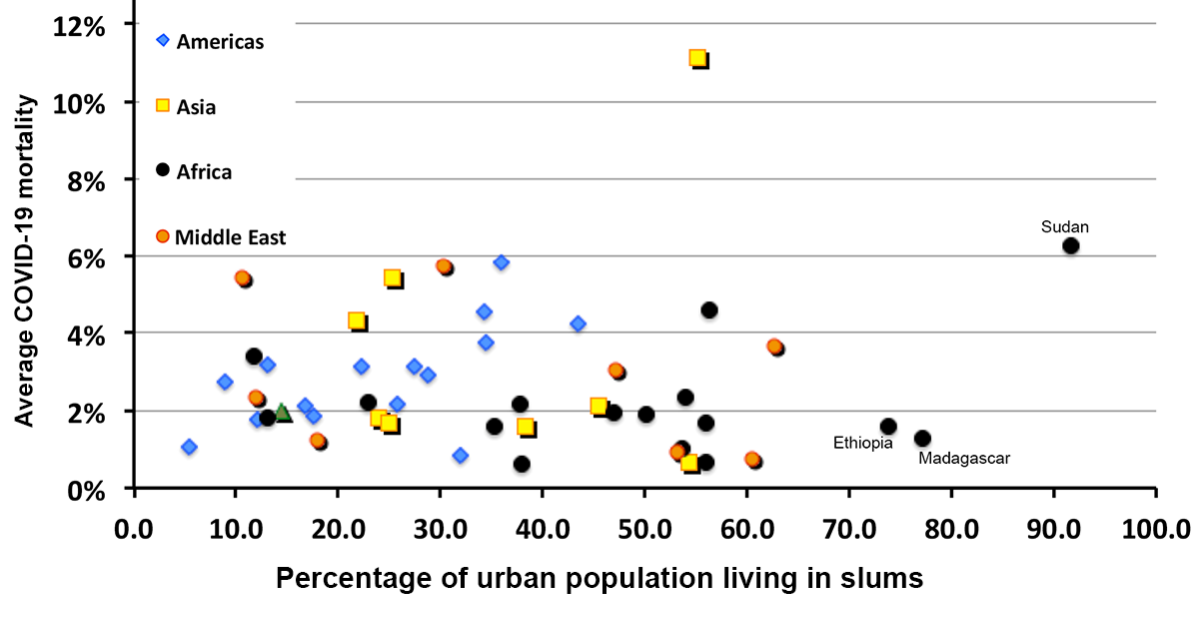
Correlation of COVID-19 mortality with the percentage of the urban population living in slums. The 3 outlying nations are identified.

The correlation with respect to GDP is explained by the correlation of the GDP with the percentage of the population aged over 65 years. The substantial correlation of contagion with testing results from the obvious fact that the more one looks, the more one sees. The correlation of contagion with the percentage of urban population is due to the cross-correlation of GDP with percentage of urban population (0.648) and the high cross-correlation of urban population with testing for COVID-19 (0.497). The values for average health care expenditures and the UHC index of the WHO are similarly explained. The data that underlie the value of case fatality rate versus the percentage of the urban population that live in slums appears in Figure 18.

## Discussion

Although this study covering statistics from countries with approximately 70% of the world’s population confirms the early clinical observation that infection with SARS-CoV-2 presents an increased risk to persons over the age of 65 years, it does not support the suggestions offered by government agencies early in the pandemic that the risks are much greater for persons with certain common potential comorbidities. Many of the early deaths of older patients early in the pandemic occurred in circumstances that likely promoted rather than impeded contagion among persons already in a generally poor state of health, likely accompanied by compromised immune functions.

Reference [7] and the analysis of Koff and Williams [16] provide plausible explanations for these findings. Namely, the virulence of COVID-19 in older people is strongly driven by the decrease in adaptive and innate immune responses with aging. Koff and Williams recommend that more longitudinal studies be performed in aging populations, including assessing the potential of a decrease in the efficacy of vaccines as being “critical to the future of global health.”

Many persons who object to strict measures to prevent the spread of SARS-CoV-2 commonly claim that COVID-19 is similar to influenza, is only slightly more lethal, and should be treated in the same manner as influenza as a matter of public policy. In fact, comparing the severity of medical outcomes of COVID-19 with those caused by influenza strains and their resulting pneumonias displays dramatic differences. Promulgating the idea that COVID-19 is a “flu-like disease” spreads gross misinformation, to the detriment of public health worldwide.

A broader comparative assessment of SARS-CoV-2 and influenza strains against an overall measure of immune system responsiveness to infection would require a global database of an appropriate metric. One potential candidate is the “Wellness Index” proposed by J Han [17]; however, that metric would require genetic sequencing of large, representative samples of individuals over a broad range of countries. Consequently, at present, the possibility that such a database of immune system readiness will be generated seems highly doubtful.

Governmental actions can reduce the consequences of SARS-CoV-2 infection. Comparing the cases of Germany and Italy may be instructive in this regard. By mid-October 2020, Italy had 150% of the numbers of confirmed cases of COVID-19 in Germany [10]. However, the case fatality rate in Italy was roughly triple that in Germany. In early 2020, Germany had established an extensive network of triage and early treatment centers outside of hospitals. Germany also moved quickly to secure adequate supplies of personal protective equipment [18]. Hence, infected patients were identified early in the course of the disease and were treated in a manner that did not overwhelm the central intensive care facilities in hospitals, as happened in the Italian region of Lombardy.

A similar lesson may come from comparing the experience in the United States in California and New York through the fall of 2020. The early lockdown in California more than doubled the duration of the first wave of infections compared with New York, leading to 60% more cases in California; however, the death rate in California was half that in the State of New York, where medical resources were badly stressed [10].

At the data cutoff date of this study, authoritative statistics on a worldwide, country-to-country basis were not publicly available to evaluate the effectiveness of either prevention or treatment modalities. However, clinical trials of multiple vaccines had been completed with highly promising results. Also unavailable over the full range of those countries included in this analysis are the full range of statistics related to COVID-19 disaggregated with respect to sex. When and if such data become available, expanding the analysis with respect to sex-based differences in testing, contagion, and mortality would prove useful.

The rollout of large-scale vaccination programs during a time when the vaccines are in short supply necessitates schemes for prioritizing recipients. If probability of severe illness is a primary consideration, then the early guess about the risks connected with potential comorbidities should be replaced with data such as those presented here along with detailed clinical evaluations accumulated throughout 2020.

**Figure 19:**
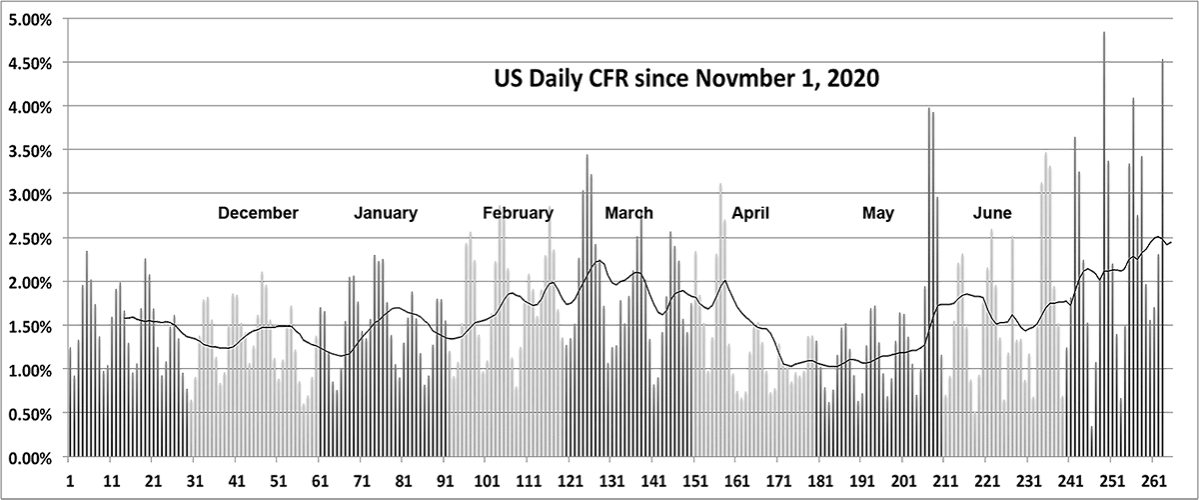
The apparent daily case fatality rate in the United States, showing a disturbing increasing trend after the appearance of the B.1.1.7 and B.1.617 strains. CFR: case fatality rate.

A word of caution: Data used in this study were accumulated before the variants of concern of SARS-CoV-2, B.1.1.7, B.1.351, P.1 and B.1.617 began to propagate. Initial evidence suggests that these new strains are somewhat more virulent than the original strain. Examining the national CFR averaged over the duration of the pandemic during early 2021 shows a troubling slight but statistically significant increase in several countries, including the United States. Indeed, based on [10], over the period from November 1, 2020, to June 18, 2021, the apparent case fatality rate in the United States looks significantly higher (Figure 19) than that before the appearance of the B.1.1.7 and B.1.617 strains. Similar behavior is observed in the data from Germany and Canada.

Admittedly, these recent data are much noisier than earlier data through all of 2020 due to the marked decrease in the daily reports of the number of new cases and deaths. Despite the reduced statistical significance, the trend is troubling. It is too soon to judge whether the increase is a reflection of increased virulence in variants of SARS-CoV-2, whether it is an indication of increased susceptibility and physical and psychological stress on so-called essential workers, or whether it is a result of some form of COVID-19–related weariness among large portions of national populations. Differences in virulence of the several variant strains now circulating will complicate the interpretation of national data collected in 2021.

## Appendix

Multimedia Appendix 1 Regional variation of COVID-19 case fatality rate correlations.

## Abbreviations

CDC: US Centers for Disease Control and Prevention
DM: diabetes mellitus
GDP-PPP: gross domestic product–purchasing power parity
UHC: Universal Health Coverage
WHO: World Health Organization
