# Supplementary material for: Risk Factors of SARS-CoV-2 Infection: Global Epidemiological Study
Source: JMIRx Med. 2021 Aug 26;2(3):e28843. doi: 10.2196/28843 (PMC8404263; doi:10.2196/28843)
Supplement: Multimedia Appendix 1 [file xmed_v2i3e28843_app1.docx]

Table S1. Regional variation of COVID-19 case fatality rate correlations.

| Factor | Region | | | | | |
| --- | --- | --- | --- | --- | --- | --- |
|  | World | Americas | Asia | Europe | Africa | Middle East |
|  | | | | | | |
| Kidney disease | 0.269 | 0.197 | –0.08 | 0.156 | 0.283 | 0.459 |
| Household size | 0.225 | 0.421 | –0.05 | 0.086 | 0.422 | 0.209 |
| Heart disease | 0.204 | –0.157 | 0.057 | –0.142 | 0.39 | 0.55 |
| Asthma | 0.165 | –0.233 | 0.038 | –0.209 | 0.177 | 0.691 |
| Diabetes deaths | 0.133 | 0.559 | 0.097 | 0.204 | –0.04 | –0.101 |
| COVID-19 deaths/1M | 0.092 | 0.38 | 0.065 | 0.726 | 0.077 | –0.246 |
| % slums | 0.09 | 0.237 | –0.421 | 0 | 0.291 | 0.227 |
| Hypertension | 0.051 | –0.048 | 0.387 | 0.101 | –0.134 | 0.024 |
| Influenza/pneumonia | 0.034 | 0.502 | –0.312 | 0.074 | –0.384 | 0.626 |
| Malnutrition | 0.017 | 0.469 | 0.289 | –0.058 | –0.154 | 0.63 |
| Lung disease | 0.013 | 0.013 | 0.124 | 0.209 | 0.414 | 0.756 |
| Random number | 0.009 | –0.214 | –0.158 | 0.269 | 0.072 | –0.132 |
| Diabetes % of population | 0.009 | 0.195 | –0.023 | –0.011 | 0.753 | –0.457 |
| Population | –0.013 | 0.029 | 0.128 | 0.108 | –0.122 | 0.16 |
| Obesity % of population | –0.017 | –0.175 | 0.059 | 0.241 | –0.012 | –0.411 |
| % of population aged ≥65 years | –0.081 | –0.212 | 0.021 | 0.192 | 0.191 | –0.042 |
| COVID-19 cases/1M | –0.153 | –0.126 | –0.343 | 0.131 | –0.053 | –0.471 |
| Health expenditures | –0.155 | –0.234 | –0.079 | –0.125 | –0.028 | –0.457 |
| Lung cancers | –0.159 | –0.405 | 0.231 | 0.270 | –0.026 | –0.347 |
| Life expectancy | –0.163 | –0.185 | –0.101 | 0.138 | 0.062 | –0.608 |
| Median age | –0.179 | –0.259 | 0.075 | 0.141 | 0.028 | –0.611 |
| World Health Organization Universal Health Coverage index | –0.197 | 0.058 | –0.029 | –0.1 | –0.034 | –0.659 |
| % of population in cities | –0.197 | –0.199 | –0.105 | –0.057 | 0.002 | –0.573 |
| Adjusted gross domestic product | –0.230 | –0.223 | –0.3 | –0.107 | 0.125 | –0.438 |
| COVID-19 tests | –0.287 | –0.069 | –0.276 | –0.324 | –0.389 | –0.498 |
